# The mechanism of NK cell expression of RANKL/RANK/OPG pathway in mouse models of femoral head necrosis

**DOI:** 10.1097/MS9.0000000000003615

**Published:** 2025-07-18

**Authors:** Junwei Dai, Jun Tao, Shusheng Wei, Baokun Han, Shuai Ma, Chengyu Wu, Long Sun, Xiaodong Ma, Yimeng Chen

**Affiliations:** a Department of Orthopedic, The First Hospital of Anhui University of Science and Technology, Huainan, Anhui, P.R. China; b Department of Orthopedic, The First people Hospital of Wuhu, Wuhu, Anhui, P.R. China

**Keywords:** NK cells, ONFH, OPG, RANK, RANKL

## Abstract

This study aimed to investigate the role of natural killer (NK) cells in the RANKL/RANK/OPG pathway in osteonecrosis of the femoral head (ONFH). C57 mice were categorized into a control group, an observation group (10 mice each), and an experimental group comprising 4 NK cell knockout mice. A hormone-induced femoral head necrosis model was created by administering lipopolysaccharide combined with methylprednisolone for 8 weeks to the experimental and control groups. The observation group received subcutaneous injections of an equal amount of normal saline. After 8 weeks, peripheral blood was collected from the mice, and bilateral femoral head specimens were obtained post-mortem. Expression levels of NK cells, OPG, RANK, and RANKL in the peripheral blood and joint fluid of ONFH mice were determined using PCR and ELISA techniques, and compared with the control group. The experimental group exhibited an increased number of NK cells in the peripheral blood and joint fluid compared to the control group. OPG expression was downregulated, while RANK and RANKL were significantly upregulated, resulting in a marked increase in the number of mature osteoclasts. In ONFH patients, NK cells were found to upregulate TNF-α and RANKL, downregulate IFN-γ and OPG, promote osteoclast maturation, disrupt bone balance, accelerate femoral head necrosis collapse, and ultimately hasten the progression of femoral head necrosis.

## Introduction

Osteonecrosis of the femoral head (ONFH) is a prevalent and challenging disease significantly impacting patients’ mobility and quality of life. It typically results from necrosis of femoral head cells, leading to bone collapse and hip joint damage^[1]^. ONFH patients often experience severe hip pain and even joint dysfunction, resulting in a high disability rate. In China, the annual incidence is approximately 100 000 people. The classic pathological process of ONFH is involves bone necrosis, bone absorption, and new bone formation^[2]^. During the bone repair process, there is an imbalance between osteoblasts and osteoclasts, with osteoclasts being more numerous and active than osteoblasts, leading to femoral head collapse^[3]^.

Both immune cells and bone cells originate from bone marrow hematopoietic stem cells, influencing bone production through different levels of cytokines in the RANKL/RANK/OPG pathway. This influence is associated with certain cytokines^[4]^. Previous studies have identified a correlation between T and B cells and ONFH, suggesting a role in the development of ONFH^[5]^. According to research, natural killer (NK) cells are involved in the pathological and physiological processes of bone diseases. For example, NK cells have been shown to regulate the formation of osteoclasts, which are cells responsible for bone resorption^[6]^. However, the role of NK cells in the mechanism of ONFH in the RANKL/RANK/OPG pathway remains unexplored.

Therefore, investigating the changes in the number of NK cells and the expression levels of related factors in the peripheral blood between the experimental group and the control group of ONFH, as well as determining the differences in the expression of the RANKL/RANK/OPG pathway, can reveal the mechanism of action of NK cells in the RANKL/RANK/OPG pathway in ONFH. This research fills a gap in current medical immunology regarding the role of NK cells in the RANK/RANKL/OPG pathway and establishes a new foundation for the diagnosis and treatment of ONFH.

With the application of AI in the medical field, new perspectives and tools have been provided for the research and treatment of ONFH. Especially machine learning and deep learning can extract valuable information from complex medical data to assist in diagnosis, prediction, and treatment decision-making^[7,8]^. Combining with the mechanism of action of NK cells in ONFH can provide new possibilities for precision medicine. By analyzing the immune status and disease characteristics of patients through AI, the activity of NK cells can be precisely regulated to optimize treatment efficacy. The development of new treatment strategies, such as enhancing the anti-inflammatory and pro angiogenic abilities of NK cells through gene editing techniques (such as CRISPR/Cas9), or developing AI based NK cell expansion and activation schemes^[9]^, as well as AI providing real-time decision support for clinical doctors, such as analyzing patients’ immune markers and imaging data to recommend the best NK cell treatment plan or combination therapy^[10]^. Nowadays, clinical images can be used to analyze genomes and diseases through AI models. If we clarify the regulation of NK cells on the process of femoral head necrosis, we can also use AI to construct corresponding models and quickly and effectively provide precise customized treatment for patients with femoral head necrosis, laying a certain foundation for our treatment of femoral head necrosis^[11]^.

## Materials and methods

The work has been fully compliant with the ARRIVE criteria^[12]^ and the TITAN Guidelines 2025^[13]^.

### Animals

Twenty sexually mature male mice, aged 6–8 weeks, along with four NK cell knockout mice cultured using Cre loxP technology which aged 6–8 weeks selected for this study. The NK cell knockout mice were designated as the experimental group, while the remaining mice were randomly divided into control and observation groups, with ten mice in each group. The age and gender of the three groups are at the same stage, used to minimize potential confounding factors

### Reagents

#### PCR equipment required

Total RNA extraction kit (Solarbio, Cat No: R1200), universal reverse transcription kit (Yeasen, Cat No: 11141ES60), Real-time PCR fluorescence quantitative kit (Yeasen, Cat No: 11201ES08),

#### Femoral head necrosis modeling equipment

injection grade methylprednisolone sodium succinate (Huabang Pharmaceutical, Cat No: 230072212), and lipopolysaccharide (Sigma, Cat No: L8880).

### Equipment

#### PCR equipment

Desktop low-speed centrifuge (Shanghai Medical Instruments, Instrument Model: 80-2), conventional PCR instrument (Tianlong, Model: Genesy 98T), real-time fluorescence quantitative PCR instrument (Molarray, Instrument Model: MA-6000), and a nucleic acid detector (Lifereal, Model: F-1100).

### Research plan and experimental methods

#### Mouse grouping

Twenty male mice aged 6–8 weeks were selected, including NK cell-deficient mice. The NK cell-deficient mice, were assigned to the experimental group, while the remaining mice were randomly assigned to the control and observation groups, with ten mice in each group.

**HIGHLIGHTS**

- Clarify the differences in NK cells between NONFH patients and healthy individuals.
- Clarify the differences in the expression levels of cytokines IFN-γ, TNF-α, and RANKL/RANK/OPG pathways between NONFH patients and healthy individuals, and identify that these differences are caused by NK cells.
- Explore the association between cytokines IFN-γ, TNF-α, and RANKL/RANK/OPG pathways and femoral head collapse, and explore new strategies for targeted NK cell therapy for NONFH.

#### Mouse modeling and sampling

The experimental group and control group mice were intraperitoneally injected with lipopolysaccharide 20 μ g/kg once a day for 2 consecutive days; On the third day, 100 mg/kg methylprednisolone was injected into the gluteal muscle for two consecutive weeks; Starting from the third week, hormone injections will be given every other day until samples are collected. The observation group was injected with physiological saline as a control, and the model was created for a total of 8 weeks. The observation group received subcutaneous injections of equal amounts of physiological saline. After 8 weeks, peripheral blood was extracted from the three groups of mice, followed by sacrificing the mice to obtain bilateral femoral head specimens.

#### Fluorescence quantitative PCR

##### Direct RNA extraction

Extract 1 ml of peripheral blood and joint fluid from the femoral heads of experimental group and control group, and add them to 1.5 ml EP tubes respectively. For peripheral blood samples, perform red blood cell lysis by centrifuging at 1500 rpm for 5 minutes, then collect the precipitate. Add 1 ml of TRIzol reagent to each tube, shake well, and allow digestion in a sterile hood for 3 minutes. Transfer the digested cell lysate to a 1.5 ml EP tube treated with DEPC, Add 0.2 ml of fresh chloroform, shake slowly for 15 seconds, and let stand at room temperature for 3 minutes. Centrifuge at 12 000 rpm for 15 minutes at 4°C. Transfer the colorless aqueous phase (~0.6 ml) to a DEPC-treated EP tube, add 0.5 ml of freshly opened isopropanol, and let stand at room temperature for 10 minutes. Centrifuge at 12 000 rpm for 10 minutes at 4°C. Observe the white precipitate of total RNA at the tube’s bottom, discard the supernatant, and wash with 1.0 ml of 75% ethanol (prepared with DEPC water),). Centrifuge at 7500 rpm for 5 minutes at 4°C. Discard the supernatant, air-dry, and remove the remaining liquid with a small pipette tip. Air-dry the precipitate for 5–10 minutes, add 20–30 μl of DEPC-treated water, mix well, and dissolve the total RNA in a 55–60°C water bath for 10 minutes.

##### Reverse transcription

In a 1.5 ml EP tube, add 10 μl of DEPC water, 1 μl of primer, 1 μl of template RNA, and 1 μl of dNTP . Briefly centrifuge, then heat at 65°C for 5 minutes. Immediately transfer to an ice-water bath for at least 1 minute. After brief centrifugation, on ice:, add 4 μl of 5 × First-Strand Buffer, 1 μl of 0.1 M DTT, 1 μl of RNase Inhibitor, and 1 μl of SSIII reverse transcriptase. Briefly centrifuge, and perform reverse transcription at 50°C for 60 minutes. Inactivate the reverse transcriptase at 70°C for 15 minutes to obtain cDNA from peripheral blood and joint fluid cells.

##### Fluorescence quantitative PCR

Add 5 μl of SYBR Premix Ex Taq, 0.2 μl of upstream primer, 0.2 μl of downstream primer, 0.5 μl of template cDNA, and 4.1 μl ofddH22O to the PCR reaction tube. Set the reaction conditions were set as follows: 95°C for 20 seconds, 55°C for 20 seconds, and 72°C for 20 seconds, for a total of 40 cycles. Perform differential analysis on the expression levels of cytokines IFN-γ, TNF-α, and the RANKL/RANK/OPG pathway. The gene primer sequences are shown in Table 1.

**Table 1:** The gene primer sequences

Cytokines	Primer sequence		Primer fragment size (bp)	Amplification fragment size (bp)
GAPDH	F	AGAAGGTGGTGAAGCAGGCATCT	23	117
	R	CGGCATCGAAGGTGGAAGAGTG	22	
TNFα	F	GGAACTGGCAGAAGAGGCACTC	22	89
	R	GCAGGAATGAGAAGAGGCTGAGAC	24	
IFNγ	F	AACTCAAGTGGCATAGATGTGGAAGA	26	171
	R	AATGACGCTTATGTTGTTGCTGATGG	26	
RANK (Tnfrsf11a)	F	CGACTGGTTCACTGCTCCTAATCC	24	271
	R	CTGCCTGTGTAGCCATCTGTTGAG	24	
RANKL (Tnfsf11)	F	GATGGAAGGCTCATGGTTGGATGTG	25	82
	R	GGCAGCATTGATGGTGAGGTGTG	23	

#### Data analysis

Quantitative analysis was performed using the 2-△△Ct method (Livak method). First, normalize the Ct values of the target gene to the Ct value of the reference gene for all test samples and calibration samples (△Ct(test) = Ct(target, test) − Ct(ref, test), △Ct(calibrator) = Ct(target, calibrator) − Ct(ref, calibrator)). Then, normalize the △Ct value of the calibration sample to the △Ct value of the test sample: △△Ct = △Ct(test) − △Ct(calibrator). Finally, calculate the expression ratio: 2-△△Ct = 2 to the power of△△Ct representing the relative expression level.

#### All quantitative data are expressed as mean ± standard deviation

Data analysis was performed using SPSS 22.0 software, and all data were tested using t-tests. Results with *P* <0.05 were considered statistically significant.

### Pathological examination of femoral head tissue

After euthanizing the rats, isolate the femoral heads, decalcify in a 10% ethylenediaminetetraacetic acid solution for 8 weeks, dehydrate in ethanol gradients, fix in xylene, embed in paraffin, section at 5 μm, stain with hematoxylin and eosin (HE), and examine histopathologically under an optical microscope.

## Results

### Clinical observation results of mouse model femoral heads

During the experiment, the control group animals maintained normal food intake, normal fur color, and consistent weight. In the observation group, animals exhibited reduced food intake, sparse hair at the femoral head site, slight weight gain, swelling at the injection site, and a limping gait. The experimental group showed similar symptoms to the observation group, but the onset of these symptoms occurred earlier in the experimental group.

### Pathological examination results of mouse model femoral heads

**Figure 1.**
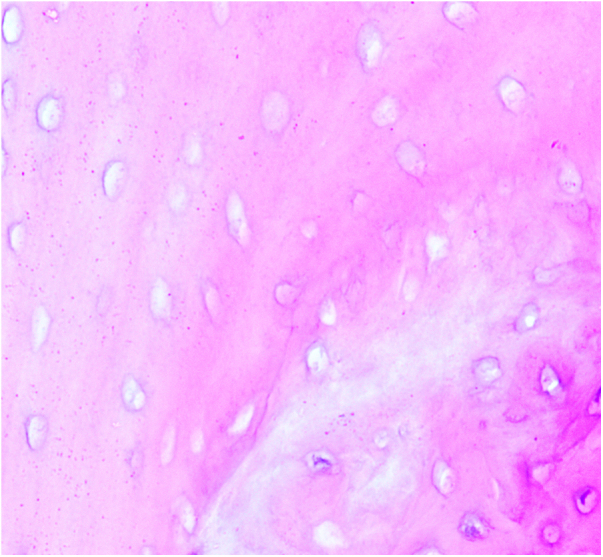
The experimental group.

**Figure 2.**
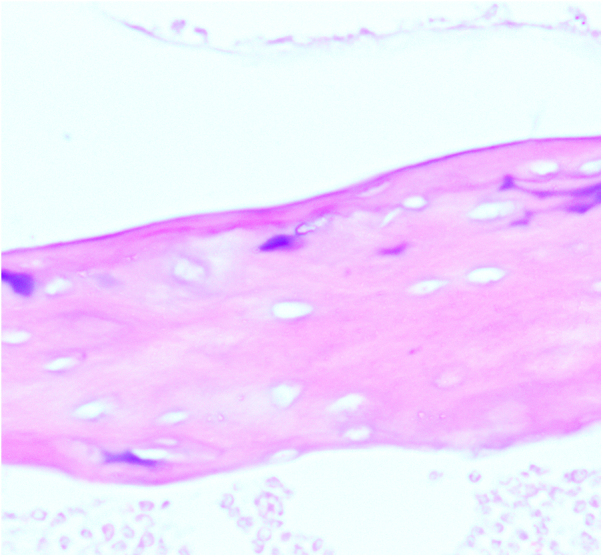
The observation group.

**Figure 3.**
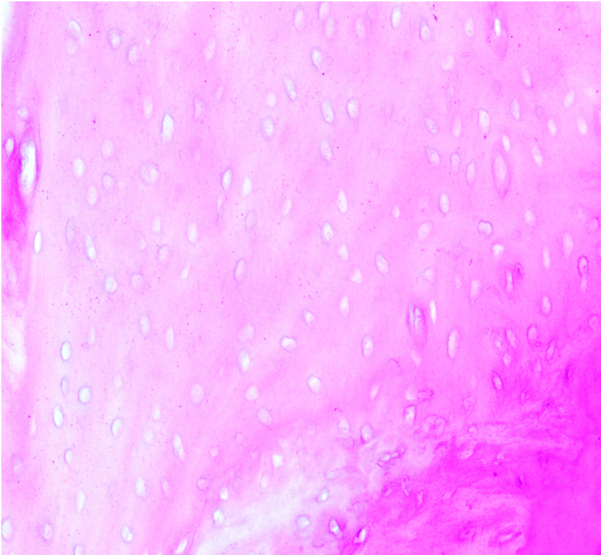
The control group.

The subchondral region of the femoral head was selected as the primary observation area. In the experimental group, signs of bone marrow tissue necrosis, condensation or even disappearance of osteocyte nuclei, and the formation of empty bone lacunae were observed. The observation group showed similar pathological features, but the extent of osteocyte lysis, condensation, and disappearance of osteocyte nuclei was less severe compared to the experimental group. In contrast, the control group exhibited healthy bone marrow tissue, with intact osteocytes, no rupture, no condensation phenomenon, and intact osteocyte nuclei Figure1, Figure 2 and Figure 3.

### Mouse model of femoral head necrosis: results of immunological examination

#### Expression of TNF-α and IFN-γ

**Figure 4.**
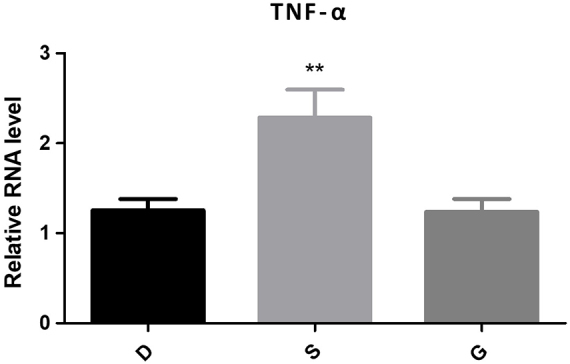
The expression levels of TNF-α of PCR.

**Figure 5.**
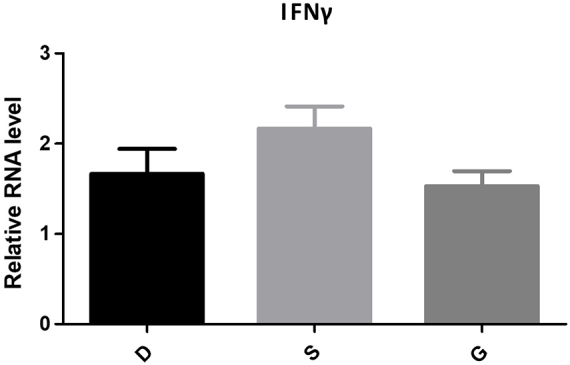
The expression levels of IFN-γ of PCR

PCR technology was used to determine the expression levels of TNF-α and IFN-γ in peripheral blood. The experimental group showed significantly higher expression of TNF-α and IFN-γ compared to the control group (*P* < 0.05). Similarly, the observation group also exhibited significantly elevated levels of TNF-α and IFN-γ compared to the control group (*P* < 0.05), with results similar to those of the experimental group. Detailed results can be found in Table 2 and Figures 4 and 5.

**Table 2:** Comparison of TNF-α and IFN-γ in peripheral blood specimens (x̄ ± s).

Grouping	n	TNF-α (Ct)	IFN-γ (Ct)
Experimental group	4	27.93 ± 1.80	26.41 ± 2.00
Control group	10	28.1 ± 1.63	26.46 ± 2.18
Observation group	10	28.45 ± 2.13	26.41 ± 1.93
t (experimental group) *P*		4.137	2.031
		<0.05	<0.05
t (observation group) *P*		5.213	2.025
		<0.05	<0.05

t represents significance in SPSS, *P* represents the degree to which the original hypothesis is not rejected, and if it is less than 0.05, it indicates a significant difference between the two groups.

#### Expression of RANKL/RANK/OPG

**Figure 6.**
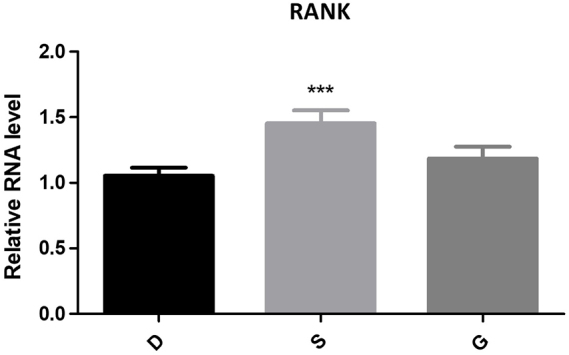
The expression of RANK of PCR.

**Figure 7.**
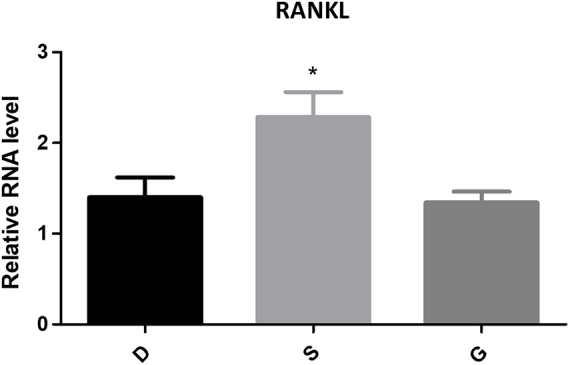
The expression of RANKL of PCR.

**Figure 8.**
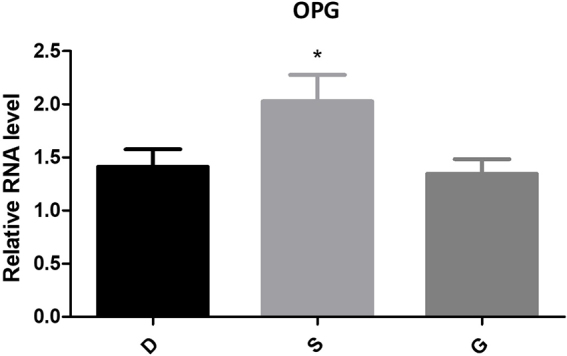
The expression of OPG of PCR.

Using PCR technology to compare the expression of OPG/, RANK/, and RANKL in the peripheral blood of the three groups, it was found that OPG expression in the experimental group was significantly lower than in the control group (*P* < 0.05). In contrast, the expression of RANK and RANKL was significantly higher than in the control group (*P* < 0.05). Similar results were observed in the observation group, where OPG expression decreased and RANK and RANKL expressions increased compared to the control group. However, t-test analysis of OPG and RANK expression levels in the observation group, yielded *P* values greater than 0.05, indicating no statistical significance. Refer to Table 3 and Figures 6–8 for detailed results.

**Table 3:** Comparison of RANKL, RANK, and OPG results in peripheral blood specimens (x̄ ± s)

Grouping	n	RANK (Ct)	OPG (Ct)	RANKL(Ct)
Experimental group	4	32.49 ± 0.49	26.01 ± 1.61	25.60 ± 2.20
	10			
Control group	10	32.37 ± 0.74	26.28 ± 1.67	25.46 ± 1.90
Observation group		32.36 ± 0.80	26.15 ± 1.27	25.73 ± 2.35
t (experimental group) *P*		1.588	1.606	2.879
		<0.05	<0.05	<0.05
t (observation group) *P*		−0.346	0.831	3.186
		>0.05	>0.05	<0.05

## Discussion

### Significance and necessity of the study

Osteonecrosis of the femoral head (ONFH) is a prevalent and challenging condition in clinical orthopedics presenting significant difficulties in early diagnosis and treatment. This disease adversely impacts patients’ mobility and quality of life while increasing healthcare costs and economic burden. The cytokines IFN-γ, TNF-α, and the RANKL/RANK/OPG pathway play roles in the reconstruction of bone cell necrosis, but their specific mechanisms in ONFH are not well understood. Extensive literature review indicates that NK cells express cytokines IFN-γ, TNF-α, and the RANKL/RANK/OPG pathway suggesting their involvement in the process of femoral head necrosis and collapse.in ONFH patients^[5]^.

This study demonstrates, through in vitro experiments, that there are significant differences in the levels of cytokines IFN-γ, TNF-α, and the RANKL/RANK/OPG pathway between ONFH patients and healthy individuals. Using a hormone-induced femoral head necrosis mouse model, we have established the correlation between these differences and NK cells. This research reveals for the first time the role of NK cells in the occurrence and progression of ONFH, In clinical practice, HBOT is a safe and effective method for treating ONFH patients. The beneficial effect of HBOT may be to regulate the OPG/RANK/RANKL pathway by upregulating the production of serum OPG, which affects the bone remodeling process of ONFH^[6]^. Our experimental results indicate that NK cells also participate in the process of femoral head necrosis by regulating the OPG/RANK/RANKL pathway. Studies have shown that chimeric antigen receptor (CAR)-NK cell therapy is a promising strategy that combines the natural cytotoxicity of NK cells with the targeted specificity of CAR. CAR-NK cells can be genetically engineered to express CARs targeting specific tumor antigens, thereby enhancing their targeting and killing abilities^[14]^. Although this strategy is currently mostly used for the treatment of tumors and immune system diseases, the pathophysiology of ONFH involves multiple factors, including vascular damage, genetic susceptibility, and inflammation. However, there is evidence to suggest that immune cells, including macrophages and neutrophils, play a role in the progression of the disease^[15]^. This opens up new ideas and provides new insights for future targeted therapy or immune stem cell transplantation.

Since their discovery, natural killer (NK) cells have been extensively studied^[16]^. They can be categorized into two subgroups based on different surface markers: CD56Bright and CD56Dim^[17]^. In peripheral blood, the CD56DimCD16Bright subgroup predominates (about 90% of total NK cells), whereas CD56BrightCD16 ± NK cells are mainly found in secondary lymphoid organs (about 10% of total NK cells). CD56Bright NK cells are capable of producing large amounts of cytokines such as interferon-γ (IFN-γ) and tumor necrosis factor α (TNF-α)^[8]^.

The theory of lipid metabolism disorder in femoral head necrosis suggests that the fundamental cause of the disease lies in the abnormal differentiation of MSCs. Studies have suggested that the concentration of glucocorticoids is the main influencing factor of MSCs differentiation direction. Lower concentration levels of glucocorticoids can induce MSCs differentiation and become bone cells, promoting rapid maturation of bone cells. Relatively high concentrations of glucocorticoids can lead to MSCs differentiation into adipocytes. High doses of glucocorticoids can cause excessive proliferation of adipocytes, leading to increased pressure in the femur and compression of microvessels, further enhancing the ischemic manifestations of femoral head necrosis. This is mainly reflected in the fact that glucocorticoids regulate the expression of OPG, TNF-α, and B receptor activation factors, thereby affecting bone metabolism^[18]^. Our experiment demonstrated that NK cells regulate OPG, RANK/RANKL, TNF-α, and other cytokines, demonstrating their promoting effect on osteoblasts and osteoclasts, thereby regulating the progression of femoral head necrosis.

Recent research utilizing collagen-induced arthritis (CIA) mouse models has revealed that NK cells have the potential to transform into osteoclasts and directly or indirectly participate in the development of osteoarthritis^[19–21]^. We summarize that in ONFH patients, NK cells upregulate TNF-α and RANKL, downregulate IFN-γ and OPG, promote osteoclast maturation, disrupt bone balance, and accelerate femoral head collapse.

### Mechanism of NK cell expression of RANKL/RANK/OPG pathway in ONFH

#### IFN-γ inhibits osteoclast maturation and participates in bone repair and reconstruction

IFN-γ isa critical immunoregulatory cytokine in the immune response, belonging to the interferon family, which includes type I, type II, and type III interferons. Recent studies have highlighted the significant role of IFN-γ in the differentiation and maturation of osteoblasts and osteoclasts. Immune cells such as B cells, pro-inflammatory M1 macrophages, and NK cells inhibit osteoclast formation by increasing IFN-γ expression^[22–24]^. Additionally, various T cells can elevate IFN-γ levels to suppress osteoclast formation, while anti-IFN-γ antibodies can block these effects in culture systems^[25]^. Ruiz *et al* reported that human osteoblast-like cells express low levels of IFN-γ^[26]^. Tian *et al* found that promotes osteoclast formation and upregulates the expression of osteoclast-specific mRNA and proteins^[27]^. Moreover, IFN-γ can promote osteoblast formation by upregulating osteogenic factors such as Runx2, osterix, Alp, and osteocalcin^[28]^.

Therefore, IFN-γ can reduce bone resorption by inhibiting the generation and function of osteoclasts. Research has shown that IFN-γ inhibits the differentiation and activation of osteoclasts by interfering with the RANKL signaling pathway. In addition, IFN-γ can induce osteoclast apoptosis, thereby further reducing bone resorption^[29]^.

On the other hand, IFN-γ also has a certain impact on bone formation. IFN-γ can enhance bone formation by promoting the differentiation and function of osteoblasts. For example, IFN-γ can stimulate osteoblasts to secrete bone matrix proteins such as osteocalcin and type I collagen, thereby promoting bone mineralization. However, the effect of IFN-γ is not entirely positive, and at high concentrations, it may have a negative impact on bone formation by inducing osteoblast apoptosis or inhibiting its function^[9]^.

Therefore, IFN-γ plays a complex dual role in bone remodeling, which depends on multiple factors including concentration, other cytokines in the microenvironment, and the signaling pathway status of target cells. On the one hand, IFN-γ can reduce bone resorption by inhibiting the generation and activity of osteoclasts; On the other hand, it can also increase bone formation by promoting the function of osteoblasts. But obviously, our experimental results indicate that during the process of femoral head necrosis, the regulation of IFN-γ by NK cells is often negative

#### TNF-α participates in the mechanism of bone repair and reconstruction in the skeletal system

Macrophage colony-stimulating factor (M-CSF) and receptor activator of nuclear factor kappa-B ligand (RANKL) are crucial factors in osteoclast differentiation^[30,31]^.

M-CSF is known to promote the survival and proliferation of osteoclast precursors, thereby upregulating the expression of RANK^[32,33]^ .Tumor necrosis factor-alpha (TNF-α) is considered a significant stimulatory factor in osteoclast differentiation. TNF-α can increase the number of local and systemic tartrate-resistant acid phosphatase (TRAP) positive osteoclasts in wild-type (WT) mice deficient in RANKL by activating the NF-κB signaling pathway^[34–36]^. Studies have shown that treating bone marrow macrophages (BMMs) at the osteoclast precursor stage with TNF-α promotes osteoclastogenesis^[37]^.TNF-α is believed to play a key role in promoting the differentiation of mature hematopoietic stem cells into osteoclasts, by increasing the levels of RANK and RANKL in macrophages and stem cells. This stimulates the binding of RANKL to its receptor RANK, activating multiple signaling pathways regulated by RANKL, such as MAPK, TRAF-2, c-jun, JNK, NF-κB, and AP-1, which mediate osteoclast proliferation and differentiation^[38–40]^. Additionally, TNF-α induces TRAF3 autophagic degradation and upregulates TRAF6-/- osteoclast precursor maturation through RANKL stimulation. Furthermore, RANKL can promote TNF-α-induced osteoclastogenesis via a TRAF6-independent signaling pathway^[41]^. However, the regulation of TNF-α by NK cells is not unidirectional. Under negative feedback regulation and partial inhibition of cytokine expression^[42,43]^, NK cells can suppress the bone resorption promoting effect of TNF-α by secreting other cytokines such as IFN-γ^[29]^. However, our results indicate that during the process of femoral head necrosis, NK cells regulate TNF positively. Therefore, TNF-α also plays an important role in bone remodeling and repair in the skeletal system.

#### RANKL/RANK/OPG pathway and bone repair and reconstruction

The receptor activator of NF-κB (RANK) is primarily expressed in osteoclast precursors, mature osteoclasts, and immune cells. When RANK binds to its ligand, the receptor activator of NF-κB ligand (RANKL), it promotes osteoclast differentiation and maturation. Osteoprotegerin (OPG) pregulating the OPG/RANKL ratio can prevent osteoclast formation^[44,45]^. Mature osteoclasts secrete vesicular RANK, which triggers a reverse signaling of RANKL by binding to RANKL on osteoblasts, thereby promoting bone formation^[46]^. Bone mass loss depends on the RANK-RANKL-OPG system, which is the main regulatory system for the induction, activation, and survival of osteoclasts^[47]^. Studies have found that IL-20 can regulate the occurrence of osteoclasts in bone marrow mesenchymal stem cells by activating the OPG/RANKL/RANK axis and the NF-κB, MAPK, and AKT pathways. This makes targeting IL-20 a promising direction for the targeted regulation of bone loss-related diseases^[48]^. These findings suggest that the RANKL/RANK/OPG pathway is crucially linked to bone repair and reconstruction.

### Mechanism integration process description

#### Inducing factors

The inducing factors of ONFH, such as glucocorticoids, may lead to abnormal NK cell activity.

#### NK cell dysfunction

Dysfunctional NK cells may secrete excessive TNF-α or reduce the secretion of IFN-γ.

#### RANKL/RANK/OPG axis imbalance

TNF-α upregulates the expression of RANKL and downregulates the expression of OPG, leading to an increase in the RANKL/OPG ratio.

#### Overactivation of osteoclasts:

Enhanced RANKL/RANK binding promotes osteoclastogenesis and bone resorption.

#### Inhibition of bone formation

Osteoblast activity may be inhibited by TNF-α or unable to function effectively due to bone matrix destruction.

#### Osteonecrosis

Bone resorption exceeds bone formation, leading to structural damage and ultimately developing into femoral head necrosis.

### Limitations of the study

#### Small sample size

This study has limitations, including the small number of mice in the experimental group, and the inability to adequately match the gender and age of the experimental, control, and observation groups, which could introduce bias. However, all mice have shown positive results through experiments, which we believe is beneficial for our conclusion judgment. in future research, we aim to use gene chip technology to test cell factors such as IFN-γ, TNF-α, RANKL, RANK, and OPG in a larger sample size. In future research, we aim to use gene chip technology to test cell factors such as IFN-γ, TNF-α, RANKL, RANK, and OPG in a larger sample size.

#### Limitations of hormone induced models

The hormone induced model has certain application value in studying immune system function, especially NK cell activity. However, this model has some significant limitations.

Physiological relevance: Hormone induced models typically simulate the hormonal environment in the body through exogenous hormones such as estrogen and androgen, but this approach may not fully replicate the hormonal fluctuations and complex regulatory networks under physiological conditions.

Short term effects: Hormone induced models typically focus on short-term effects, while the function and activity of NK cells may be affected by long-term hormone exposure.

Nonspecific effects: Hormones not only affect NK cells, but may also have a broad impact on other immune cells such as T cells and B cells, thereby interfering with the interpretation of experimental results.

#### Potential bias of NK cell knockout model

This model has some potential biases and limitations:

Compensation mechanism: After NK cell knockout, other immune cells (such as T cells and macrophages) may partially replace the function of NK cells through compensation mechanism, thereby masking the true role of NK cells.

The influence of experimental conditions: The experimental results of NK cell knockout models may be affected by experimental conditions (such as cell culture conditions, selection of animal models).

In future research, we aim to use gene chip technology to test cell factors such as IFN-γ, TNF-α, RANKL, RANK, and OPG in a larger sample size. And repeat the experimental results multiple times to reduce bias

## Conclusion

In summary, our results indicate a significant increase in the proportion of NK cells in patients with ONFH and in animal models. Within the RANK/RANKL/OPG system, OPG expression is significantly downregulated, while RANK and RANKL expression is upregulated This promotes osteoclast maturation, disrupts bone balance, increases bone cell death and resorption, and accelerates femoral head collapse. From Our study suggests that NK cells play a role in accelerating the disease progression in ONFH patients Consequently, targeting NK cells to reduce their cytotoxic activity, or upregulating OPG while downregulating RANK and RANKL expression, could inhibit osteoclast maturation. This approach may help prevent bone cell necrosis and resorption in the femoral head, ultimately delaying femoral head necrosis, collapse, and deformation.

## Data Availability

All data that support the findings of this study are included in this manuscript and its supplementary information files.

## Research registration unique identifying number (UIN)

This paper belongs to animal research and does not involve human experiments or data.
